# Early age at first sexual intercourse and early pregnancy are risk factors for cervical cancer in developing countries

**DOI:** 10.1038/sj.bjc.6604974

**Published:** 2009-03-10

**Authors:** K S Louie, S de Sanjose, M Diaz, X Castellsagué, R Herrero, C J Meijer, K Shah, S Franceschi, N Muñoz, F X Bosch

**Affiliations:** 1 Unit of Infections and Cancer, Cancer Epidemiology Research Program, Catalan Institute of Oncology, Hospitalet del Llobregat (Barcelona), Avda. Gran Via s/n Km 2,7, Barcelona 080907, Spain; 2 CIBER en Epidemiología y Salud Pública (CIBERESP), Barcelona, Spain; 3 Proyecto Epidemiológico Guanacaste, Fundación INCIENSA, Torre La Sabana, 300 Oeste del ICE, Piso 7, Sabana Norte, San José, Costa Rica; 4 Department of Pathology, VY University Medical Center, PO Box 7057, 1007 MB Amsterdam, The Netherlands; 5 Department of Molecular Microbiology and Immunology, Johns Hopkins Bloomberg School of Public Health, Baltimore, MD, USA; 6 International Agency for Research on Cancer, 150 cours Albert Thomas, 69372 Lyon cedex 08, France; 7 Instituto Nacional de Cancerologia, Bogotá, Colombia

**Keywords:** cervical cancer, first sexual intercourse, pregnancy, sexual behaviour, child sexual abuse

## Abstract

Early age at first sexual intercourse (AFSI) has long been associated with an increased risk of invasive cervical carcinoma (ICC). Age at first pregnancy (AFP) and ICC have been investigated less, although AFSI and AFP are strongly interrelated in most developing countries. A pooled analysis of case–control studies on ICC from eight developing countries with 1864 cases and 1719 controls investigated the roles of AFSI, AFP, and ICC risk. Age at first sexual intercourse, AFP and age at first marriage (AFM) were highly interrelated and had similar ICC risk estimates. Compared with women with AFSI ⩾21 years, the odds ratio (OR) of ICC was 1.80 (95% CI: 1.50–2.39) among women with AFSI 17–20 years and 2.31 (95% CI: 1.85–2.87) for AFSI ⩽16 years (*P*-trend <0.001). No statistical interaction was detected between AFSI and any established risk factors for ICC. The ICC risk was 2.4-fold among those who reported AFSI and AFP at ⩽16 years compared with those with AFSI and AFP at ⩾21 years. These data confirm AFSI and AFB as risk factors for ICC in eight developing countries, but any independent effects of these two events could not be distinguished.

Early age at first sexual intercourse (AFSI) has been associated with an increased risk of high-risk human papillomavirus (HPV) infection, a sexually transmitted infection (STIS), that in susceptible women is responsible for virtually all cases of invasive cervical cancer (ICC) (Bosch *et al*, 2002). As sexual behaviour determines exposure to HPV, AFSI is of particular interest as it has been associated with riskier sexual behaviour, such as having unprotected sex, having multiple sexual partners, as well as a woman's partner having multiple partners. It has also been speculated that the increased risk of HPV is because of a biological predisposition of the immature cervix during adolescence that may be more susceptible to persistent HPV infections and therefore have a greater risk of cancer development (Kjaer *et al*, 1998). A number of studies have identified an increased risk of high-grade lesions and/or cervical cancer with early AFSI, whereas others have not (IARC, 2007). However, many of these studies were conducted before HPV assessment was feasible, and therefore, the association remains inconclusive. Age at first marriage (AFM) is often used as a proxy measure for AFSI, and those who engage in early sexual intercourse may also consequently become pregnant at an early age. Besides early AFSI, early childbearing has also been linked as a risk factor for cervical carcinogenesis and attributed to the cervical trauma experienced during early age at first pregnancy (AFP), or subsequently, by high-parity births (IARC, 2007). The interpretation of the mechanisms by which these sexual and reproductive events occurring early in life might affect ICC risk three or more decades later is not straightforward. The objective of this study is to further characterise and provide robust estimates of the risk of cervical cancer and its association with AFSI, interrelated characteristics such as AFP and AFM in a series of studies that fully considered the association of HPV with cervical cancer.

## Materials and methods

The programme of HPV and cervical cancer studies has been coordinated by the International Agency for Research on Cancer (IARC) in Lyon, France and the Institut Català d'Oncologia (ICO) in Barcelona, Spain. They included a series of case-control studies on ICC from eight developing countries with a broad range of rates of incidence of cervical cancer that were pooled for analysis. Regions covered include Morocco (Chaouki *et al*, 1998) and Algeria (Hammouda *et al*, 2005) in Africa; the Philippines (Ngelangel *et al*, 1998), Thailand (Chichareon *et al*, 1998) and Madras in Asia; and Brazil (Eluf-Neto *et al*, 1994), Colombia (Munoz *et al*, 1993), Paraguay (Rolon *et al*, 2000) and Peru (Eluf-Neto *et al*, 1994) in South America. Although Spain (Munoz *et al*, 1993) was part of the series of case–control studies, the sexual and reproductive behaviour of this population was heterogeneous to the other countries (late AFSI and low parity) and the study site was therefore excluded from this analysis.

The methods of each study have been described elsewhere. Briefly, women with histologically confirmed incident invasive squamous cell carcinoma (SCC), adenocarcinoma or adenosquamous-cell carcinoma were recruited from reference hospitals before treatment. Written informed consent was obtained from those who agreed to participate. Hospital-based controls were frequency-matched to case patients by 5-year age groups.

A standardised questionnaire was administered to the participants by a trained interviewer, which included questions about sociodemographic factors, sexual and reproductive behaviour, smoking habits, pap screening history, hygienic practices, and history of sexually transmitted diseases.

Two samples of cervical exfoliated cells were collected with wooden spatulae and endocervical brushes. After preparation of one Papanicolaou smear, the remaining cells were eluded in saline, centrifuged and frozen at −70°C until shipment to the central laboratory for HPV DNA testing. A tumor-biopsy sample was obtained from cases and frozen. Cytology and histology diagnoses were reviewed and confirmed by a panel of expert pathologists that agreed on a diagnosis by consensus or majority.

Detailed descriptions of the polymerase-chain-reaction (PCR) assays used in these studies have been described elsewhere. HPV DNA detection was detected by PCR amplification of a small fragment of the *L1* gene using MY09 and MY11 consensus primers for the study in Colombia (Hildesheim *et al*, 1994) and the GP5+/6+ general primer system for the other studies (Walboomers *et al*, 1992; Jacobs *et al*, 1995; Roda Husman *et al*, 1995). β-Globin primers were used to amplify the *β*-globin gene to assess the quality of the DNA in the specimen. HPV DNA in PCR products was analysed using a cocktail of HPV-specific probes and genotyped by hybridisation with type-specific probes for 33 HPV types. Samples that tested positive for HPV DNA but did not hybridise with any of the type-specific probes were labelled as HPV X.

### Statistical analysis

Unconditional logistic regression was used to estimate odds ratios (ORs) and 95% confidence intervals (95% CI). To assess the association of AFSI with the risk of ICC, three different statistical models to adjust for HPV DNA detection were computed and compared: (1) one model included all patients and controls, and it was not adjusted for HPV DNA status, (2) a second model included all patients and controls, and included a variable to adjust for HPV DNA status, and (3) a third model was restricted to HPV–DNA-positive cases and controls. To control for potential confounding, final models were adjusted for age (<40, ⩾40), country, lifetime number of sexual partners (1,>1), parity (0, 1–4,⩾5), and educational level (never, primary, secondary or higher). Each variable included in the adjustment models was assessed for interaction with AFSI. Test for trend was carried out when appropriate, using the log-likelihood-ratio test. Only subjects who reported ever having been married and/or ever having had children were included in the analyses of AFM and AFP.

We evaluated other potential confounding factors such as smoking (never, ever), oral contraceptive use (never, 1–4 years, ⩾5 years), history of pap smears excluding those in the 12 months before enrolment (never, ever), having had first sexual intercourse before menarche and the timing of first sexual intercourse relative to age at menarche (data not shown), but they were not adjusted for in the final analysis as they did not contribute any change to the OR estimates for AFSI in the adjusted models.

## Results

Table 1 describes some characteristics of the 1864 ICC cases and 1719 corresponding controls that entered the final analysis. Ninety-five percent of case patients and 17% of controls tested positive for HPV DNA. The majority of cases (92%) had SCC. Case patients were older than controls with a median age of 49 *vs* 48, respectively. Median AFSI was earlier in case patients (17 years) compared with controls (19 years), and this was found to be consistent in each country.

Table 2 shows the risk of ICC by AFSI according to the three different adjustment models. An increased risk of ICC was consistently observed with decreasing AFSI (*P*-trend <0.001). Compared with AFSI ⩾21 years, the OR of ICC was 1.80 (95% CI: 1.50–2.16) for AFSI 17–20 years, and 2.31 (95% CI: 1.85–2.87) for AFSI ⩽16 years, after adjusting for age, centre, lifetime number of partners, parity, and education level in the HPV-unadjusted model. According to the different model adjustments, women reporting AFSI ⩽16 years of age had a 2.3–2.5-fold risk of ICC and 1.8–2.1-fold risk for AFSI 17–20 years of age (Table 2). Given the consistent association of AFSI and the risk of ICC across the different models, HPV-unadjusted models were used for the remainder of the results.

We calculated the risk of ICC for each country study, and, in general, each study showed an increasing risk of ICC with decreasing AFSI (data not shown). There was no evidence of heterogeneity with respect to study country (*P*=0.58).

We stratified the analysis according to the established risk factors for ICC and the positive association of ICC with decreasing AFSI remained at each level of exposure for each of these characteristics (Table 3). Similar associations were observed for AFP. No interaction was observed between any of the examined risk factors and AFSI. Although not statistically significant, the risk linked to AFSI seemed to be stronger among parous women compared with nulliparous women.

**Figure 1:**
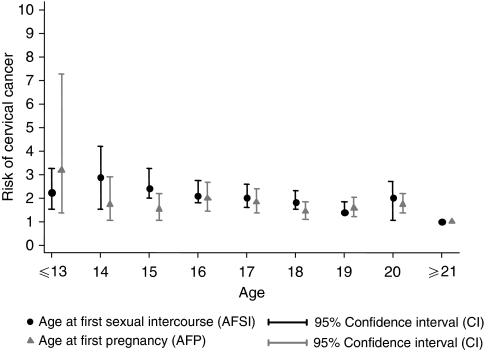
Age at first sexual intercourse and age at first pregnancy are highly correlated with the risk of invasive cervical cancer (*P*-trend<0.001). Models were adjusted for age, centre, lifetime number of sexual partners (1, >1), parity (0, 1–4, ⩾5), and education (never, primary, secondary).

Age at first pregnancy and AFM were both directly correlated with AFSI in these populations (*P*<0.001). Approximately, 92% of women reported AFSI to be the same as AFM. One-quarter of women reported AFP to be the same as AFSI. Cumulatively, 62.4% of women reported giving birth within the first year of AFSI. Among women with AFSI ⩽16 years, 52.4% were pregnant within the first year of sexual intercourse. Figure 1 shows the high correlation between AFSI and AFP, and the similar decreasing risk of ICC with increasing age of AFSI/AFP. Given the high correlation between the two variables, we did not adjust for AFSI in the AFP final model and vice versa.

We further evaluated the combined effect of AFP and AFSI on the risk of cervical cancer (Table 4). An increased risk emerged in subsequent strata of decreasing AFP with decreasing AFSI. Given this combined effect, we assessed the latency period (AFP−AFSI) between these two events to clarify whether it affected the cervical cancer risk. Although there was no statistical difference across strata, the data suggested that within each AFSI strata, women with a latency period for a subsequent pregnancy of <2 years may be at a slight increased risk compared with women with a larger time gap (data not shown).

## Discussion

The IARC/ICO series of case–control studies remain the largest set of aetiological investigations on ICC that fully addresses the role of HPV DNA and of the independent established cofactors. This is probably also the largest dataset reporting on ICC in the developing world in which early AFSI, AFP and high parity are prevalent phenomenons. The results show that early AFSI and early AFP are risk factors for cervical cancer, irrespective of other known risk factors for the disease. The data presented show a possible additional increase in risk when the early event of first sexual intercourse is shortly followed by a pregnancy.

The mechanism by which the early experience of first sexual intercourse and first pregnancy could influence the risk of cervical carcinogenesis may be explained by the steroid hormonal influence on HPV infection and on the host's immune response to HPV during pre-adolescence and adolescence. The transformation zone of the cervical epithelium has been recognised as the site in which HPV infection tends to cause cancer, and the susceptibility of this area is believed to be related to its denudation of the stratified epithelium, thus facilitating exposure of the basal layer to HPV with minimal trauma. Biological immaturity during adolescence has also been proposed as an additional susceptibility factor (Moscicki *et al*, 1989; Elson *et al*, 2000; Singer and Monaghan, 2000). During adolescence and pregnancy, the cervix is exposed to augmented levels of hormonal changes (Singer and Monaghan, 2000), in which oestrogen stimulation facilitates acidification of the vaginal cavity, a determinant of squamous metaplasia when the endocervical epithelial everts (Elson *et al*, 2000). When this oestrogen-stimulated metaplastic transformation occurs in the presence of HPV, the probability of cell transformation increases, resulting in neoplastic changes (Elson *et al*, 2000; Shai *et al*, 2007, 2008; Hwang *et al*, 2009). This phenomenon is dependent primarily on parity, and is more likely to occur during the first pregnancy rather than subsequent pregnancies (Singer and Monaghan, 2000). Although it has been postulated that these metaplastic changes are also influenced by the trauma and repair experienced during delivery, no increased risk for cervical carcinoma was observed in this same dataset when traumatic partition was evaluated (Munoz *et al*, 2002).

Increased risks of cervical carcinoma have been identified in women with long-term use of hormonal steroids (Moreno *et al*, 2002) and those who are highly parous (Munoz *et al*, 2002). In addition, HPV-16 transgenic mouse models have shown that those treated with longer durations of oestrogen were more likely to develop larger tumours and have a significantly higher number of tumours than those treated with a shorter duration (Elson *et al*, 2000; Brake and Lambert, 2005), supporting the human observations of a susceptible cervix to carcinogenic progression by continuous exogenous oestrogen exposure or increased endogenous oestrogen levels. If indeed oestrogen is needed for cervical carcinogenesis, close follow-up of young women and of their early pregnancies may be relevant to further understanding the role of steroids in the acquisition and persistence of HPV infections.

The influence of oestrogens on immune response may offer another explanatory effect (Mitrani-Rosenbaum *et al*, 1989; Arbeit *et al*, 1996), particularly during the follicular phase of the ovarian cycle and pregnancy, when levels of oestrogens are increased up to 3–8-fold the normal levels (Duncan *et al*, 1994; Marzi *et al*, 1996; Jabbour *et al*, 2008). The higher density of oestrogen receptors and their expression in the transformation zone may synergise with the effects of HPV oncoproteins, decreasing levels of cytotoxic cytokines that may down-regulate the cervical cell-mediated immune response, which favour persistent HPV infections instead of clearance (Marzi *et al*, 1996; Giannini *et al*, 1998; 2002; Jacobs *et al*, 2003). Additional research is needed to further understand the interaction between oestrogens and the regulation of immunomodulators, which may contribute to anti-tumour immunity.

The varying results between studies regarding the roles of AFSI and AFP may reflect the true differences between the study populations. In our study, the similar increased risks shown for AFSI and AFP may, in general, reflect the fact that in most developing countries women initiate these events at an early age, and experience high parity, making their effects difficult to distinguish from one another. In contrast, results of studies in more developed countries where there is a longer latency period between sexual initiation and AFP, as in Spain, the US (Brinton *et al*, 1987) or Italy (Parazzini *et al*, 1989) tend to show an increased risk with early AFSI but not with AFP as first pregnancies tend to occur much later. It is interesting that, in countries like the UK, where the rates of teenage pregnancies are high, women with AFSI of ⩽17 years had a 2–3-fold increased risk for cervical cancer compared with those with AFSI ⩾20 years (Green *et al*, 2003). Consistently, women with an early AFP of 15–19 years had a two-fold increased risk for cervical cancer compared with those with AFP ⩾25 years (Green *et al*, 2003). These observations merit further exploration but, in aggregate, tend to indicate a significant increase in risk of neoplastic disease when early AFSI occurs (surrogate of early HPV exposure and a period of increased cervical susceptibility) and is followed closely by an early pregnancy (surrogate of early exposure to high oestrogen levels).

Irrespective of their lifetime number of sexual partners, women have a similar increased risk of ICC with early AFSI as shown by the 2.4-fold risk among monogamous women with AFSI ⩽16 years as compared with the 2.2-fold risk among women with >1 lifetime number of sexual partners. It has long been suggested that a cervical cancer risk will also depend on the sexual history of the woman's male partner in addition to her own behaviour (Skegg *et al*, 1982). This is particularly relevant in societies where most women are virgins at marriage and monogamous thereafter, where the incidence of cervical cancer for a population may vary depending on the behaviour of the male partner. Of our study women, 70% were monogamous. In several studies among monogamous women, the risk of cervical cancer was reported to be two to eight times for women with husbands who had multiple partners (Pridan and Lilienfeld, 1971; Buckley *et al*, 1981; Brinton *et al*, 1989). The sexual history of the male partner was not evaluated in this analysis; however, promiscuity, history of other STIS, and lack of male circumcision are factors that have been associated with the male role in cervical carcinogenesis (Castellsague *et al*, 2003).

In interpreting our results, we must emphasise the difficulty in fully disentangling a woman's sexual and reproductive profile in relation to her cancer risk (Schroder *et al*, 2003). We cannot exclude misclassification bias if AFSI and the number of sexual partners were inaccurately reported, leading to some residual confounding, However, the presence of established risk factors for ICC, use of oral contraceptives, smoking, and pap smear history did not seem to significantly affect the strength of the association between AFSI, AFP, and risk of ICC.

We examined the different stratified methodologies (unadjusted, HPV-adjusted, and HPV-positive restricted) used to evaluate the association between AFSI and risk of ICC traditionally employed in the literature. This was done to exclude any spurious association related to statistical adjustment and to clarify inconsistent findings of the association found in earlier studies. Although in strict terms restriction of analyses to HPV-positive cases and controls seemed preferable, the consistency of the results across the three different methods provides convincing evidence of the risk associated with AFSI. Furthermore, these results indicate that for the evaluation of other risk factors, adjusting for HPV status is not necessary as the adjustments do not contribute to remove any confounding effect.

Sexual practices in the world indicate that very early intercourse might be occurring in adolescents with 44, 45 and 52% of girls between the ages of 13–19 years reporting being sexually experienced in Argentina, Botswana and Nigeria, respectively (Brown *et al*, 2001). In several case studies among young females, first sexual intercourse has been reported as forced in 5–15% of cases, and in some extreme cases worldwide, the estimates range from 21% among out-of-school adolescents in Botswana, 20% among secondary schools in Peru, and 41% among young urban females attending night schools in Peru. Among 15–30% of sexually active girls aged 15–19 years report forced first sexual intercourse (Brown *et al*, 2001). It is likely that the partners of these adolescents who report sexual coercion are adult males who are sexually experienced (Wellings *et al*, 2006) and at high risk of HPV exposure. Globally, these exposures might affect a high proportion of very young girls in areas of human strife, thus adding child sexual abuse to the burden of a lifetime increased risk of genital cancer.

Our study shows that women who initiate first sexual intercourse and experience their first pregnancy at a young age are at an increased risk of cervical cancer. The importance of HPV-vaccination programmes targeting young adolescents before first sexual intercourse can have a great effect in decreasing the incidence of cervical cancer; additional efforts are required in family planning and sexual education adapted to the extremely variable sociocultural contexts in the world.

## Figures and Tables

**Table 1:** Characteristics of cases of invasive cervix carcinoma and controls

	**HPV tested**		**HPV positive**				**Age^a^**		**Age at sexual debut^a^**	
	**Cases**	**Controls**	**Cases**	**%**	**Controls**	**%**	**Cases**	**Controls**	**Cases**	**Controls**
Country	1864	1719	1769	94.9	285	16.6	49	48	17	19
Algeria	142	145	132	93.0	18	12.4	53.5	52	16	18
Morocco	188	176	182	96.8	38	21.6	49	40	16	18
Madras (India)	187	184	180	96.3	51	27.7	48	46.5	17	18
Philippines	364	380	349	95.9	35	9.2	47.5	47	19	21
Thailand	378	259	363	96.0	41	15.8	49.5	50	18	20
Brazil	187	190	181	96.8	32	16.8	51	52	18	19
Colombia	110	124	87	79.1	21	16.9	46	45.5	17	18
Paraguay	112	86	109	97.3	18	20.9	48.5	45.5	16	19
Peru	196	175	186	94.9	31	17.7	48	48	16	18

Abbreviation: HPV=human papillomavirus.

a Median.

**Table 2:** Effect of different strategies of multivariate model adjustments on the association between age at first sexual intercourse and risk of ICC (from IARC case–control studies)

			**Age and centre adjusted**	**HPV unadjusted^a^**	**HPV adjusted^a^**	**HPV-positive only^a^**
**Sexual debut**	**Cases *n* (%)**	**Controls *n* (%)**	**Odds ratio (95% CI)**	**Odds ratio (95% CI)**	**Odds ratio (95% CI)**	**Odds ratio (95% CI)**
⩾21 years	341 (16.9)	656 (35.4)	1.00	1.00	1.00	1.00
17–20 years	813 (40.2)	667 (36.0)	2.44 (1.07–2.87)	1.80 (1.50–2.16)	1.78 (1.32–2.39)	2.10 (1.49–2.97)
⩽16 years	710 (35.1)	396 (21.4)	4.09 (3.38–4.94)	2.31 (1.85–2.87)	2.09 (1.48–2.96)	2.48 (1.65–3.73)
*P-trend*				<0.001	<0.001	<0.001

Abbreviations: HPV=human papillomavirus; ICC=invasive cervical carcinoma; IARC=International Agency for Research on Cancer; CI=confidence interval.

a Adjusted for age, study country, lifetime number of partners (1, ⩾2), parity (0, 1–4, ⩾5), and education (never, primary, secondary).

**Table 3:** Age at first sexual intercourse and risk of cervical cancer according to various characteristics

	**Number of** **cases/controls**	**Odds ratio** **(95% CI)^a^**	**Odds ratio** **(95% CI)^b^**	***P*-trend**
*Parity*				
*Nulliparous*				
⩾21 years	14/40	1.00	1.00	
17–20 years	7/14	1.88 (0.53–6.66)	1.61 (0.40–6.57)	
⩽16 years	7/6	3.60 (0.83–15.52)	1.50 (0.17–13.55)	0.56
*Ever parous*				
⩾21 years	327/614	1.00	1.00	
17–20 years	804/645	2.47 (2.07–2.94)	1.97 (1.63–2.36)	
⩽16 years	703/387	4.10 (3.36–4.99)	2.59 (2.08–3.21)	<0.001
				
*P-heterogeneity between AFSI and ever parous=0.64*				
Parity (1–4 births)				
⩾21 years	171/399	1.00	1.00	
17–20 years	273/287	2.58 (1.98–3.35)	1.99 (1.51–2.62)	
⩽16 years	169/115	4.72 (3.41–6.54)	2.71 (1.89–3.87)	<0.001
*Parity (⩾5 births)*				
⩾21 years	156/215	1.00	1.00	
17–20 years	531/358	2.01 (1.57–2.59)	1.71 (1.32–2.23)	
⩽16 years	534/272	2.88 (2.19–3.78)	2.08 (1.55–2.78)	<0.001
*P-heterogeneity between AFSI and parous groups (nulliparous, 1–4 births, and ⩾5 births)=0.90*				
				
*Oral contraceptive use*				
*Never*				
⩾21 years	218/400	1.00	1.00	
17–20 years	453/364	2.33 (1.87–2.90)	1.77 (1.40–2.25)	
⩽16 years	376/184	4.28 (3.29–5.55)	2.45 (1.82–3.29)	<0.001
*1–4 years*				
⩾21 years	64/162	1.00	1.00	
17–20 years	117/114	2.64 (1.76–3.94)	1.67 (1.07–2.61)	
⩽16 years	111/89	3.94 (2.53–6.13)	1.95 (1.15–3.30)	0.01
*⩾5 years*				
⩾21 years	36/52	1.00	1.00	
17–20 years	124/72	3.26 (1.85–5.72)	2.46 (1.36–4.46)	
⩽16 years	96/49	4.48 (2.39–8.40)	2.80 (1.38–5.65)	0.006
				
*Smoking*				
*Never*				
⩾21 years	272/569	1.00	1.00	
17–20 years	587/552	2.34 (1.93–2.83)	1.68 (1.36–2.06)	
⩽16 years	550/348	3.76 (3.03–4.67)	2.06 (1.61–2.64)	<0.001
* Ever*				
⩾21 years	67/85	1.00	1.00	
17–20 years	221/113	2.73 (1.81–4.13)	2.32 (1.50–3.60)	
⩽16 years	152/43	5.63 (3.42–9.27)	3.62 (2.10–6.26)	<0.001
				
*Lifetime number of sexual partners*				
*Monogamous*				
⩾21 years	270/569	1.00	1.00	
17–20 years	529/499	2.33 (1.92–2.83)	1.80 (1.46–2.22)	
⩽16 years	349/214	3.89 (3.05–4.96)	2.38 (1.83–3.11)	<0.001
* Partners >1*				
⩾21 years	69/74	1.00	1.00	
17–20 years	280/151	2.03 (1.37–3.01)	1.74 (1.15–2.63)	
⩽16 years	352/156	2.75 (1.84–4.11)	2.14 (1.39–3.28)	0.001
*P*-heterogeneity=0.36				

*Education*				
*Never go to school*				
⩾21 years	54/57	1.00	1.00	
17–20 years	255/155	1.68 (1.09–2.60)	1.46 (0.93–2.30)	
⩽16 years	402/173	2.41 (1.55–3.75)	2.09 (1.31–3.35)	0.001
* Primary school*				
⩾21 years	164/238	1.00	1.00	
17–20 years	404/311	1.99 (1.54–2.56)	1.62 (1.24–2.12)	
⩽16 years	236/167	2.51 (1.87–3.39)	1.71 (1.24–2.36)	0.001
* Secondary school*				
⩾21 years	118/359	1.00	1.00	
17–20 years	150/198	2.68 (1.95–3.68)	2.29 (1.65–3.20)	
⩽16 years	72/55	4.79 (3.07–7.47)	3.36 (2.07–5.47)	<0.001
*P*-heterogeneity=0.08				
				
*Ever have a pap smear 12 months before study enrolment*				
*Never*				
⩾21 years	183/320	1.00	1.00	
17–20 years	434/371	2.20 (1.74–2.79)	2.29 (1.65–3.20)	
⩽16 years	416/205	3.65 (2.79–4.78)	3.36 (2.07–5.47)	<0.001
*Ever*				
⩾21 years	158/336	1.00	1.00	
17–20 years	379/296	2.65 (2.02–3.46)	1.92 (1.44–2.57)	
⩽16 years	294/191	4.49 (3.32–6.06)	2.30 (1.63–3.24)	<0.001

Abbreviations: CI=confidence interval; AFSI=age at first sexual intercourse.

a Adjusted for age and study centre.

b Adjusted for age, study country, lifetime number of partners (1, ⩾2), parity (0, 1–4, ⩾5), and education (never, primary, secondary).

**Table 4:** Interaction between age at first pregnancy and age at first sexual intercourse in the risk of cervical cancer

	**Age at first pregnancy**		
**Age at sexual debut**	**⩾21**	**17–20**	**⩽16**
⩾21 years	1.00		
17–20 years	1.58 (1.22–2.03)	1.93 (1.58–2.36)	
⩽16 years	2.17 (1.35–3.47)	2.28 (1.74–2.99)	2.36 (1.82–3.07)
